# In silico p*K_a_* prediction

**DOI:** 10.1186/1758-2946-4-S1-P55

**Published:** 2012-05-01

**Authors:** Robert Körner, Iurii Sushko, Sergii Novotarskyi, Igor V Tetko

**Affiliations:** 1 eADMET GmbH, Munich, 85764, Germany; 2 Helmholtz Zentrum München, Munich, 85764, Germany

## 

The biopharmaceutical profile of a compound depends directly on the dissociation constants of its acidic and basic groups, commonly expressed as the negative decadic logarithm p*K*_a_ of the acid dissociation constant (*K*_a_). The acid dissociation constant (also protonation or ionization constant) *K*_a_ is an equilibrium constant defined as the ratio of the protonated and the deprotonated form of a compound. The p*K*_a_ value of a compound strongly influences its pharmacokinetic and biochemical properties. Its accurate estimation is therefore of great interest in areas such as biochemistry, medicinal chemistry, pharmaceutical chemistry, and drug development. Aside from the pharmaceutical industry, it also has relevance in environmental ecotoxicology, as well as the agrochemicals and specialty chemicals industries.

In literature, a vast number of different approaches for p*K*_a_ prediction can be found [1]. These approaches can be divided into two different classes. On the one hand there are direct calculations, so called ab initio methods, trying to determine the p*K*_a_ value by quantum chemical or mechanical computation. On the other hand, statistical models, trained on chemical or structural descriptors. These descriptors can be, for example, of quantum chemical, semi empirical, graph topological or simple statistical nature. This type of modeling is called QSPR (Quantitative Structure Property Relationship).

In our recent work, we develop such a QSPR model using localized molecular descriptors to train multiple linear regression and artificial neural networks to estimate dissociation constants (p*K*_a_). The performance of our approach is similar to that of a semi-empirical model based on frontier electron theory [2] as well as a prediction model based on Graph Kernels [3].

How such a prediction model can be built, is shown by an example performed with OCHEM, an *online chemical database with an environment for modeling* (http://ochem.eu/). It is a publicly accessible database for chemical compound data and predictive models. Further, users get the facility to develop, apply, and distribute predictive models, so it is unique in its combination of compound data and predictive models.
